# Perspectives in Melanoma: meeting report from the Melanoma Bridge (December 1st–3rd, 2022—Naples, Italy)

**DOI:** 10.1186/s12967-023-04325-x

**Published:** 2023-07-28

**Authors:** Paolo A. Ascierto, Sanjiv S. Agarwala, Allison Betof Warner, Marc S. Ernstoff, Bernard A. Fox, Thomas F. Gajewski, Jérôme Galon, Claus Garbe, Brian R. Gastman, Jeffrey E. Gershenwald, Pawel Kalinski, Michelle Krogsgaard, Rom S. Leidner, Roger S. Lo, Alexander M. Menzies, Olivier Michielin, Poulikos I. Poulikakos, Jeffrey S. Weber, Corrado Caracò, Iman Osman, Igor Puzanov, Magdalena Thurin

**Affiliations:** 1 Department of Melanoma, Cancer Immunotherapy and Innovative Therapy, Istituto Nazionale Tumori IRCCS “Fondazione G. Pascale”, Naples, Italy; 2 Temple University School of Medicine, Philadelphia, PA USA; 3 Stanford University School of Medicine, Stanford, CA USA; 4 ImmunoOncology Branch (IOB), Developmental Therapeutics Program, Cancer Therapy and Diagnosis Division, National Cancer Institute (NCI), National Institutes of Health (NIH), Bethesda, MD USA; 5 Robert W. Franz Cancer Center, Earle A. Chiles Research Institute, Providence Cancer Institute, Portland, OR USA; 6 Department of Pathology and Department of Medicine (Section of Hematology/Oncology), University of Chicago, Chicago, IL USA; 7 INSERM, Laboratory of Integrative Cancer Immunology, 75006 Paris, France; 8 Centre de Recherche Des Cordeliers, Sorbonne Université, Université de Paris, Paris, France; 9 Equipe Labellisée Ligue Contre le Cancer, Paris, France; 10 Center for Dermatooncology, Department of Dermatology, Eberhard Karls University, Tuebingen, Germany; 11 Department of Surgery, School of Medicine, Case Comprehensive Cancer Center, Case Western Reserve University, Cleveland, OH USA; 12 Department of Surgical Oncology, The University of Texas MD Anderson Cancer Center, Houston, TX USA; 13 Department of Immunology, Roswell Park Comprehensive Cancer Center, Buffalo, NY USA; 14 Laura and Isaac Perlmutter Cancer Center and Department of Pathology, New York University Grossman School of Medicine, New York, NY USA; 15 Earle A. Chiles Research Institute, Providence Cancer Institute, Portland, OR USA; 16 Jonsson Comprehensive Cancer Center David Geffen School of Medicine at UCLA, Los Angeles, CA USA; 17 Melanoma Institute Australia, The University of Sydney, Royal North Shore and Mater Hospitals, Sydney, Australia; 18 Department of Oncology, Geneva University Hospital, Geneva, Switzerland; 19 The Tisch Cancer Institute, Icahn School of Medicine at Mount Sinai, New York, NY USA; 20 Laura and Isaac Perlmutter Cancer Center, a NCI-Funded Comprehensive Cancer Center, NYU School of Medicine, New York, NY USA; 21 Division of Surgery of Melanoma and Skin Cancer, Istituto Nazionale Tumori “Fondazione Pascale” IRCCS, Naples, Italy; 22 Rudolf L, Baer, New York University Langone Medical Center, New York, NY USA; 23 Department of Medicine, Roswell Park Comprehensive Cancer Center, Buffalo, NY USA; 24 Division of Cancer Treatment and Diagnosis, National Cancer Institute (NCI), National Institute of Health (NIH), Bethesda, MD USA

**Keywords:** Melanoma, Immunotherapy, Anti-PD-1, Anti-CTLA-4, Target therapy, Biomarkers, BRAF inhibitor, MEK inhibitor, Adjuvant, Neoadjuvant, Combination strategies

## Abstract

Outcomes for patients with melanoma have improved over the past decade with the clinical development and approval of immunotherapies targeting immune checkpoint receptors such as programmed death-1 (PD-1), programmed death ligand 1 (PD-L1) or cytotoxic T lymphocyte antigen-4 (CTLA-4). Combinations of these checkpoint therapies with other agents are now being explored to improve outcomes and enhance benefit-risk profiles of treatment. Alternative inhibitory receptors have been identified that may be targeted for anti-tumor immune therapy, such as lymphocyte-activation gene-3 (LAG-3), as have several potential target oncogenes for molecularly targeted therapy, such as tyrosine kinase inhibitors. Unfortunately, many patients still progress and acquire resistance to immunotherapy and molecularly targeted therapies. To bypass resistance, combination treatment with immunotherapies and single or multiple TKIs have been shown to improve prognosis compared to monotherapy. The number of new combinations treatment under development for melanoma provides options for the number of patients to achieve a therapeutic benefit. Many diagnostic and prognostic assays have begun to show clinical applicability providing additional tools to optimize and individualize treatments. However, the question on the optimal algorithm of first- and later-line therapies and the search for biomarkers to guide these decisions are still under investigation. This year, the Melanoma Bridge Congress (Dec 1^st^–3rd, 2022, Naples, Italy) addressed the latest advances in melanoma research, focusing on themes of paramount importance for melanoma prevention, diagnosis and treatment. This included sessions dedicated to systems biology on immunotherapy, immunogenicity and gene expression profiling, biomarkers, and combination treatment strategies.

## Introduction

Outcomes for patients with melanoma have improved over the past decade because of the clinical development and Food and Drug Administration (FDA) approval of immunotherapies targeting checkpoint receptors such as programmed death-1 (PD-1), programmed death ligand 1 (PD-L1) or cytotoxic T lymphocyte antigen-4 (CTLA-4). However, combinations of checkpoint therapies with other checkpoint inhibitors and other agents are being explored to improve outcomes and enhance benefit-risk profiles of treatment.

PD-1 and CTLA-4 targeting therapies increase average life expectancy for cancer patients. The benefit of dual checkpoint blockade with anti CTLA-4 and anti PD-1 inhibitor over monotherapy with a CTLA-4 inhibitor has been shown, with durable disease control and improved overall survival (OS). However, PD-1 and CTLA-4 blocking agents are not effective in all patients, and even those patients who do respond initially can relapse, highlighting the need for improved treatment regimens. Alternative inhibitory receptors have been identified that may also be targeted for anti-tumor immune therapy. These include the T cell immunoglobulin and mucin-domain containing-3 (TIM-3), lymphocyte-activation gene-3 (LAG-3), TIGIT, and B-and T-lymphocyte-associated protein (BTLA) receptors associated with T cell exhaustion and V-domain immunoglobulin suppressor of T cell activation (VISTA), a receptor found on tumor-infiltrating myeloid cells.

The inhibition of two immune checkpoints, LAG-3 using relatlimab and PD-1, improved progression-free survival (PFS) to a greater extent than inhibition of PD-1 alone in patients with previously untreated metastatic or unresectable melanoma. These results support the synergistic effect of dual checkpoint inhibition over monotherapy and identify relatlimab–nivolumab as a potential new treatment option for patients with previously untreated metastatic or unresectable melanoma.

Several oncogenes have been identified as potential targets for molecularly targeted melanoma therapy, such as tyrosine kinase inhibitors (TKIs). The therapeutic effectiveness and combinatory effects were shown for agents targeting BRAF, MEK, NRAS, KRAS, HRAS, c-Kit, c-MET, VEGFR, and PI3K/AKT. Several of these therapies are already FDA-approved for treating metastatic melanoma.

Unfortunately, many patients still progress and acquires resistance to the immunotherapy and molecularly targeted therapies. To bypass resistance, combination treatment with immunotherapies and single or multiple TKIs have been shown to improve the prognosis of melanoma patients compared to monotherapy. The combination of ipilimumab and nivolumab and the combination of BRAF and MEK inhibitors have improved survival of patients and are current standards for combination therapies in immunotherapy and targeted therapy of melanoma.

For certain patients with inoperable metastatic melanoma, a triplet combination therapy may improve survival outcome. Recently a phase II study of the combination of nivolumab and dabrafenib and trametinib shows promise in treating patients with metastatic melanoma who did not respond to immunotherapy as well as melanoma patients with brain metastases.

Many diagnostic and prognostic assays have begun to show clinical applicability providing additional tools to optimize and individualize treatments. Multiple immunohistochemical biomarkers associated with the prognosis of malignant melanomas, including PD-L1, show association with clinical outcomes. Transcriptomics based assays also become technologically feasible. The example of such assay is DecisionDx (Castle Biosciences; Friendswood, Texas), that is a commercially available 31-gene expression platform, which uses 28 genes signature to be used on formalin-fixed paraffin-embedded (FFPE) tumor specimen. Panels screening multiple melanoma microRNAs or epigenetic markers in malignant melanoma have also been developed. The identification of new metabolites, antigens, and enzymes in blood that could be used as a marker of disease progression and predictors of patient outcomes is being explored.

In summary, the number of new combinations treatment under development for melanoma provides options for the number of patients to achieve a therapeutic benefit. However, the question on the optimal algorithm of first-and later-line therapies and the search for biomarkers to guide these decisions are still under investigation.

This year, the Melanoma Bridge Congress (Dec 1^st^–3rd, 2022, Naples, Italy) addressed the latest advances in melanoma research, focusing on themes of paramount importance for melanoma prevention, diagnosis and treatment. This included sessions dedicated to systems biology on immunotherapy, immunogenicity and gene expression profiling, biomarkers, and combination treatment strategies.

## Melanoma is a model for cancer research

### Combining 10 × scRNA-seq and 100 plex mIF: too much data or complimentary technologies to examine what works

Although cancer cell immunity research has been primarily T cell-centric, there is increasing evidence that B cells effector mechanisms exist and have a critical role in tumour control. These include complement-dependent cytotoxicity, antibody-dependent cellular cytotoxicity, antibody mediated apoptosis or receptor blockade, and killer B cells (Fas-FasL). The B cell response reveals antigens recognized by cytotoxic T lymphocytes (CTLs) and is part of a coordinated synergistic T and B cell immune response to cancer.

Complex vaccines in combination with costimulatory agents, e.g., anti-OX40 or anti-glucocorticoid-induced tumour necrosis factor receptor-related protein (GITR) plus anti-PD-1, can augment therapeutic efficacy. In an ongoing trial of triplet immunotherapy with vaccine plus anti-GITR and delayed anti-PD-1, tumor biopsy monitoring strategy using multiplex immunohistochemistry platform based on an exploratory 98-plex panel, single-cell T cell receptor/B cell receptor sequencing after sorting for CD45+, and RNA-sequencing are being employed to help improve understanding of the T and B cell response.

Previously, multispectral imaging revealed that the number of FoxP3 + or PD-L1+ cells within close proximity (30 μm) of CD8+ T cells to tumor cells was associated with a high level of CD8+ T cell suppressive elements and reduced OS in patients with oral squamous cell cancer, [1]. Ultrahigh-plex spatial phenotyping of proteins in FFPE tissues could be used to characterize the potential immune escape mechanisms. However, the utility of such an approach is unclear given the lack of validation. One attempt at validation could be through comparison with CITE-seq (cellular indexing of transcriptomes and epitopes), a sequencing-based method that simultaneously quantifies cell surface protein and transcriptomic data within a single cell readout. Comparison of ultrahigh-plex and single-cell RNA-sequencing plus CITE-Seq is being done in an attempt to identify gene clusters that impact on response to immunotherapy.

### Can proteomics predict both immune-related adverse events and recurrence in melanoma patients treated with immune checkpoint inhibitors?

Immune-related adverse events (irAEs) can limit the success of checkpoint inhibitor therapies. There is a critical need to identify patients at risk for severe irAEs to guide treatment selection but pre-treatment biomarkers to help predict irAEs are lacking.

Baseline serum autoantibody signatures may be able to predict recurrence and severe toxicity in patients treated with adjuvant nivolumab, ipilimumab, or nivolumab plus ipilimumab. Proteomic profiling of 300 patients using > 16,000 autoantibody-based protein array (discovery cohort) identified proteomic irAE signature. The signature was then validated in 950 patients from the CheckMate 238 and CheckMate 915 trials [2]. High recurrence score signatures predicted significantly worse recurrence-free survival (RFS) in separate nivolumab, ipilumumab, and nivolumab plus ipilimumab treated cohorts. Similarly, severe toxicity score signature was a significant predictor of severe irAEs. Predicting both, recurrence and irAEs simultaneously should better inform decision-making, and to identify patients who achieve high treatment efficacy and low irAEs. For patients predicted to have low treatment efficacy and high irAES alternative therapy options should be considered. Several autoantibody profiling platforms are available and clinical assay inter-platform robustness needs to be assessed. Development of CLIA-certified assays requires more investigation of the best technology adaptable to real world clinical care. More research is also needed to determine which autoantibodies-antigen pairs are predictive of specific irAEs, and whether autoantibody signatures are predictive of recurrence in the adjuvant setting and accurately predict response in the metastatic setting.

Another important question is whether autoantibody signatures can predict irAEs in minority populations with melanoma. Progress in melanoma treatments in the last decade has not improved outcomes for patients with melanoma from minority populations to the same extent that it has for non-Hispanic White patients. Minority populations have higher rate of autoimmune disease and may be more susceptible to developing severe irAEs after immune checkpoint inhibition. Subtypes of anti-PD-1 therapy-related irAEs in advanced melanoma vary by ethnicity, with higher rates of endocrine, liver, and other rare types of irAEs in non-White patients [3]. As such, minority melanoma patients unlike White population may have a different baseline autoimmune predisposition for developing irAEs after immune checkpoint inhibitor treatment with the irAE signature race-dependent and cancer site-agnostic. In summary, there is a need to develop a predictive signature for development of irAEs using baseline autoantibodies in minority patients with melanoma. The signatures in minority patients need to be compared to the melanoma signature in White patients as well as develop signatures for other solid tumors, such as lung or bladder cancer.

### Survivorship challenges in the immune checkpoint inhibitor era

Survivorship is becoming an important consideration among patients treated with immune checkpoint inhibitors (ICIs). Around half of all patients with metastatic cancer are potential candidates for treatment with ICIs, which can be associated with prolonged survival and a potential curative impact. There is also an increasing use of adjuvant and neoadjuvant ICI therapy for high-risk primary cancers. The extent and magnitude of ICI sequelae and chronic toxicities which impact long-term quality of life are relatively unknown. As such, there is inadequate guidance for follow-up care and management for the medical and patient communities.

Long-term OS has been observed in clinical trials with immuno-oncology agents in patients with advanced melanoma, with patients who are alive at 3 years likely to achieve prolonged cancer remission [4]. Patients with other cancers have also shown long-term survival with anti-PD-1 therapy [5, 6]. Although ICIs are generally well tolerated but have unpredictable development of moderate and severe irAEs. These can occur across multiple organ types and time to resolution can be longer than one year [7]. In patients with advanced or recurrent non-small-cell lung cancer (NSCLC) treated with nivolumab, occurrence of irAEs was positively associated with PFS and OS [8]. Potential consequences of a relationship between irAEs and response to immune checkpoint inhibition include related pathways of cancer control and pre-existing autoimmune response (acute, chronic and/or delayed toxicity) and parallel pathways with T cell populations in organ-specific toxicity, e.g., CD8 resident T cells in colitis, cytotoxic memory CD4 in encephalitis.

Long-term survivors of advanced melanoma treated with ipilimumab had overall worse health-related quality of life with more physical symptoms than matched controls [9]. Immune checkpoints help maintain health by limiting the extent of autoimmune disease generation, maintaining foetal tolerance, and limiting the inflammatory response to injury and infection. PD-1 knockout mice experience spontaneous autoimmune disease, cardiomyopathy, lupus-like glomerulonephritis, and increased mortality from graft-versus-host-like disease. These data suggest anti-PD-1 therapy can have a detrimental long-term effect on health.

In patients with metastatic renal cell carcinoma treated with interleukin (IL)-2, interferon (IFN)-α and dendritic cell (DC) vaccine, pre-treatment peripheral blood lymphocytes exhibited gene expression and serum cytokine profiles consistent with inflammation and proliferation not found in healthy donors [10]. However, there was less difference in inflammatory gene expression between patients and healthy donors after treatment. Early changes in circulating T cell repertoire are associated with increased survival in patients with advanced NSCLC after PD-L1 blockade [11].

The pathophysiology of irAEs involves the development of autoimmunity, including the release of auto-reactive T cells and generation of pre-existing auto-reactive antibodies, the on-target attack of shared tumor antigens on normal tissue, target tissue expression of immune checkpoints (e.g., CTLA-4 on normal pituitary), and inflammatory cytokine release (e.g., IL-17 and colitis). Organs at risk are those with pre-existing exposure to environmental insult (e.g., skin, lung), pre-existing autoimmunity, pre-existing genetic polymorphism associated with risks for immune-related illnesses, and microbiota, e.g., autoantibodies to *Subdoligranulum didolesgii* activated T cells in rheumatoid arthritis. The challenges in defining chronic or delayed toxicity, the correct attribution of delayed irAEs, a lack of understanding of the risk for late complications from immune checkpoint blockade, and the rarity of events. The Society of Immunotherapy for Cancer (SITC) has taken the important first steps in harmonizing and defining irAE terms and clarify the definition of chronic and re-emergent adverse events [12]. A chronic toxicity grading system, identification of toxicity modifiable pathways and risk categories, incorporation of patient-reported outcomes, development of appropriate surveillance and treatments, and providing survivorship guidelines still need to be developed. The expanding use of ICI treatments in cancer patients and their impact on prolonging overall survival is accompanied by the need to address the survivor-specific concerns by the medical community.

### Neoadjuvant trial design and clinical update in melanoma

A major challenge with the use of adjuvant therapy in melanoma is that many patients will not have disease recurrence even without treatment, others will relapse even with therapy, and only a small proportion, approximately 20%, benefit from therapy. However, all these patients are exposed to therapy that is associated with toxicity, the treatment itself, and high cost. Neoadjuvant therapy may provide an alternative option for many of these patients. The OpACIN study first showed that neoadjuvant therapy with ipilimumab and nivolumab resulted in increased T cell expansion and greater activation of anti-tumor activity than an adjuvant approach [13]. In a pooled analysis of six clinical trials with 192 patients, a pathological complete response (pCR) occurred in 40% of patients, 33% with immunotherapy and 47% with targeted therapy [14]. pCR correlated with recurrence-free survival (RFS), that differed between therapy. In patients receiving targeted therapy, pCR was required for a survival benefit, and even then around 20% had recurrence. In contrast, any response, including a pathological partial response (pPR), seemed to confer an excellent survival benefit in patients who received immunotherapy. Both high tumor mutation burden (TMB) and high IFN-γ signature score were shown associate with pathologic response and low risk of relapse [15].

In the more recent PRADO trial, therapeutic lymph node dissection (TLND) and adjuvant therapy were omitted in patients achieving major pathologic response (MPR) in their index (largest baseline) lymph node after neoadjuvant immunotherapy, whereas patients with pPR underwent TLND only, and nonresponding patients underwent TLND, adjuvant systemic therapy (nivolumab or dabrafenib/trametinib) and radiotherapy [16]. Nearly two-thirds of patients had a MPR and avoided TLND. Two-year RFS rate was 93% in patients with MPR. There was an indication of higher recurrence in patients with pPR (64%) than previously observed, and that adjuvant therapy appeared to benefit patients with no response (pNR). In another trial (DONIMI), a biomarker-driven approach showed that a high IFN-γ signature adequately identified patients who are likely to benefit from nivolumab alone compared to those with a low IFN-γ signature, for whom different treatment approaches should be considered [17]. In a subsequent trial, neoadjuvant nivolumab plus relatlimab followed by surgery and adjuvant combination therapy, resulted in a 57% pCR rate and 70% overall pathologic response rate among 30 patients [18]. The 1- and 2-year RFS rates were 100% and 92% for patients with any pathologic response.

In the first randomized phase II trial, neoadjuvant pembrolizumab led to a practice changing improvement in event-free survival (EFS) in patients with stage III–IV melanoma after a median follow-up of 14.7 months, with a a 2-year EFS of 72% compared with 49% with adjuvant pembrolizumab [19]. There is also a suggestion of improved OS although data are not mature. Several other trials are ongoing in the neoadjuvant setting, such as the phase III NADINA trial, as well as trials with intralesional agents.

Neoadjuvant immunotherapy is also being investigated in stage II disease. In the INTRIM trial, patients with stage II pT3-4/cN0 melanoma had significantly reduced tumour positive sentinel lymph node rates after presurgical single-dose treatment with the toll-like receptor-9 (TLR9) agonist IMO-2125 compared with placebo [20]. However, more patients in the placebo group had ulcerated melanoma, a known factor influencing sentinel node involvement, which precludes any firm conclusions.

### The MD Anderson Melanoma Moon Shot: a model for high-risk, high-reward research

The University of Texas MD Anderson Moon Shots Program^®^ launched in 2012 with the intent of laying a framework for a visionary new approach to research that could be applied to all cancers, and involving comprehensive, multidisciplinary teams, the identification of unmet needs and gaps in knowledge and capabilities to set project priorities, and accountability for measurable progress through goals dependent upon go/no-go milestones. In advanced melanoma, opportunities include improved understanding of mechanisms of response/resistance, novel treatment combinations for patients not responding to standard therapies, better outcomes for patients with surgically resectable disease and patients with CNS metastases, as well as prevention and early detection. At MD Anderson, the Melanoma Moon Shot goals are to reduce melanoma incidence and mortality through integrated research efforts spanning the melanoma continuum. This includes developing and delivering personalized treatment options to reduce melanoma mortality and to reduce incidence and ultimately deaths from melanoma through public policy initiatives, education, and early detection.

Melanoma prevention remains a major challenge, with integrated nationwide interventions to promote UV protection lacking in the USA. It has been established that overexposure to ultraviolet radiation increases melanoma risk. Empowering public policymakers to make informed evidence-based decisions regarding the dangers of indoor tanning has led to increased legislation in the USA; multiple states have indoor tanning bed restrictions for minors under 18 years old and there has been a reduction in the numbers of high school students using indoor tanning devices. This has provided a unique opportunity to share lessons learned and provide resources to others, including the partnering of experts from the MD Anderson Cancer Center with stakeholders in Poland leading to the prohibition of solaria use for persons < 18 years old across Poland.

Another Melanoma Moon Shot project focusses on the microbiome and host factors across the melanoma continuum. Higher diversity and different composition of the gut microbiome was observed in responders to PD-1 blockade versus non-responders [21]. Higher dietary fibre was associated with significantly improved PFS in 128 patients treated with immune checkpoint inhibitors, especially those without probiotic use [22]. The effect of a high fibre dietary intervention versus an isocaloric control diet is now being further investigated in a trial in patients with metastatic melanoma, including in adjuvant and neoadjuvant settings, and renal cell carcinoma.

Other projects are investigating novel strategies for patients with melanoma CNS metastases, personalizing early-stage disease management [e.g., through multimodal assessment of patients with high-risk stage II and sentinel lymph node biopsy (SLNB)-positive stage III melanoma] to develop improved risk models for regional and distant recurrence, and early melanoma detection. It is hoped these and other Melanoma Moon Shot research efforts will help revolutionize the conventional medical research approach and help reduce the burden of melanoma.

### Loco-regional treatments for melanoma

In a multinational analysis of 987 patients with 2482 cutaneous tumour lesions conducted by the pan-European International Network for Sharing Practice, the most frequent indications for electrochemotherapy (ECT) were basal cell carcinoma, malignant melanoma and squamous cell carcinoma, and three-quarters of patients received intravenous rather than intratumoral bleomycin [23]. The overall response rate (ORR) was 85%, with 70% complete responses, and ORR was high across all indications. A higher complete response rate was observed in patients with lesions < 3 cm diameter; for these smaller tumors, linear array electrodes provided better control than hexagonal electrodes. Intravenous administration was superior to intratumoural delivery, especially for tumours ≥ 2 cm diameter.

In a more recent study by the same network, 716 patients with either ulcerated or non-ulcerated cutaneous tumors and metastases treated with ECT were assessed [24]. Complete response rate was higher for non-ulcerated lesions than ulcerated lesions (65% vs. 51%). In smaller lesions (< 3 cm), the complete response rate was similar in ulcerated and non-ulcerated lesions (71%); responses were lower overall in lesions ≥ 3 cm, with a higher response in non-ulcerated lesions (50% vs. 33%). Patients with ulcerated lesions had higher pain and more severe symptoms compared to non-ulcerated lesions, which reduced during ECT. In patients with non-ulcered lesions, pain increased during ECT but then returned to levels similar to pre-treatment. ECT has also previously been shown to improve patients’ quality of life, with improvements in daily and social activities [25].

ECT in melanoma requires an integrated approach and can be combined with immunotherapy. This approach appears to be feasible and is well tolerated with evidence of a synergistic effect. In a retrospective analysis of 15 patients with metastatic melanoma treated with ipilimumab and ECT, local objective response was 67% and a systemic response was observed in 60% of patients [26]. Similarly, a retrospective analysis of patients with stage IIIC-IV melanoma suggested that ECT improved tumor control in patients with melanoma treated with pembrolizumab, with a higher ORR and significantly longer PFS and OS in patients treated with pembrolizumab plus ECT versus pembrolizumab alone [27].

Another locoregional approach in melanoma is the oncolytic virotherapy, talimogene laherparepvec (T-VEC). In 26 patients with early metastatic (stage IIIB/C-IVM1a) melanoma, 16 (61.5%) had a complete response and seven (26.9%) had a partial response [28]. One-fifth of patients received post T-VEC immunotherapy with pembrolizumab. In a phase 2 study of patients with unresectable stage IIIB-IVM1c malignant melanoma, T-VEC plus ipilimumab showed higher antitumor activity without additional toxicity concerns versus ipilimumab alone [29]. There was a reduction in lesion burden from baseline, with an abscopal effect on distant, non-treated lesions. In another trial, neoadjuvant T-VEC plus surgery versus upfront surgery for patients with resectable stage IIIB-IVM1a melanoma reduced the risk of disease recurrence and resulted in durable improvements in survival at 5 years [30]. These results suggest that an intratumorally administered oncolytic agent can elicit a meaningful long-term systemic effect and supports neoadjuvant T-VEC plus surgery in advanced melanoma.

## Less is more: critical issues in clinical practice

### Less is more with Immunoscore

The Immunoscore, which is based on densities of CD3+ and cytotoxic CD8+ T cells in the tumor and invasive margin, was originally validated as a predictive estimate of the risk of recurrence in patients with stage I–III colon cancer [31] and has since been included in several clinical guidelines. In stage III colon carcinoma patients, the use of Immunoscore in four independent cohorts with a total of 2514 patients have confirmed its predictive ability for patients at high-risk and no-risk of recurrence [32, 33]. Immunoscore also predicts recurrence and survival in high-risk patients with untreated early-stage colon cancer. In an evaluation of 1885 patients with stage I-II colon cancer, Immunoscore was significantly associated with survival in stage II, high-risk stage II [based on clinico-pathological high-risk features], T4N0 and microsatellite-stable (MSS) patients [34]. In stage II T4N0 colon cancer patients, Immunoscore was the only significantly predictive parameter in multivariate analysis and was the most important predictor of relapse [35].

In rectal cancer, the use of Immunoscore may help patients to avoid surgical intervention. In patients with locally advanced rectal cancer receiving neoadjuvant therapy followed by surgery, a diagnostic biopsy-adapted Immunoscore predicted response to treatment and disease-free survival [36]. In addition, no recurrence was observed in patients with high Immunoscore in a separate watch-and-wait cohort. As such, this biopsy-based Immunoscore approach could help in the selection of patients who could achieve favorable outcomes and be eligible for a watch-and-wait strategy.

The immune contexture and Immunoscore [37] may also have utility in patients with relapsed/refractory diffuse large B cell lymphoma (DLBCL) receiving chimeric antigen receptor (CAR) T cell therapy. Among 51 patients treated with axicabtagene ciloleucel, an anti-CD19 CAR T cell therapy, in the ZUMA-1 trial, clinical response and OS were associated with pre-treatment immune contexture as characterized by Immunoscore [38]. High densities of subsets of cytotoxic T-cells (CD3+ CD8+ expressing PD-1 + LAG-3 ± TIM-3) were significantly associated with response to CAR T therapy.

The Immunoscore Immune Checkpoint assay may identify patients with metastatic colorectal cancer who are likely to benefit from the addition of immune checkpoint inhibitor therapy to standard first-line treatment. In the AtezoTRIBE trial, the addition of the PD-L1 inhibitor atezolizumab to standard first-line treatment (FOLFOXIRI plus bevacizumab) improved PFS in patients with previously untreated metastatic colorectal cancer [39]. Post-hoc analyses showed a significant correlation between a high Immunoscore IC result and response to combination immunotherapy in MSS patients.

### Less is more in combination therapies

PD-1-based combination therapy represents a very active field of research in melanoma and in oncology in general. To date, there have been numerous successes (e.g., anti-PD-1 with anti-LAG-3), ineffective strategies (e.g., anti-PD-1 with an IDO inhibitor), and those potentially detrimental [e.g., anti-PD-1 with bempegaldesleukin (BEMPEG)].

Results in clinical trials of targeted therapy with BRAF/MEK inhibition plus anti-PD-1 therapy in patients with BRAF-mutant melanoma have been inconsistent. No PFS benefit was seen with the addition of spartalizumab to dabrafenib and trametinib [40] but PFS was significantly improved with the addition of atezolizumab to vemurafenib and cobimetinib [41]. Moreover, in the CheckMate 067 trial, a clinical benefit was observed numerically with nivolumab plus ipilimumab versus nivolumab alone in the general patient population, with increased benefit in patients with BRAF-mutant melanoma [42]. Nivolumab plus relatlimab provided a PFS benefit versus nivolumab alone in the RELATIVITY-047 trial in patients with previously untreated metastatic or unresectable melanoma [43]. Given these multiple combination therapy options, treatment decisions need to be individualized and consider multiple factors. For instance, data from CheckMate 067 support the use of anti-PD-1 plus anti-CTLA-4 over anti-PD-1 alone or anti-PD-1 plus anti-LAG-3 in patients with BRAF mutant melanoma. In patients with low PD-L1 expression, anti-PD-1 with either anti-CTLA-4 or anti-LAG-3 appears preferable to anti-PD-1 monotherapy. However, in patients with high PD-L1 expression, anti-PD-1 monotherapy may be more appropriate given its lower toxicity and the lack of r added benefit of a combined therapy. Anti-PD-L1 plus anti-CTLA-4 may also be a better option in patients with high lactate dehydrogenase, brain metastases, or mucosal melanoma, while anti-PD-1 plus anti-LAG-3 may be preferred in the elderly and perhaps patients with low tumour burden. PD-1 alone may be a suitable option for patients who are unfit for management of irAEs or who have desmoplastic melanoma. All these characteristics often co-exist, hence the need for an individualized strategy.

**Fig. 1:**
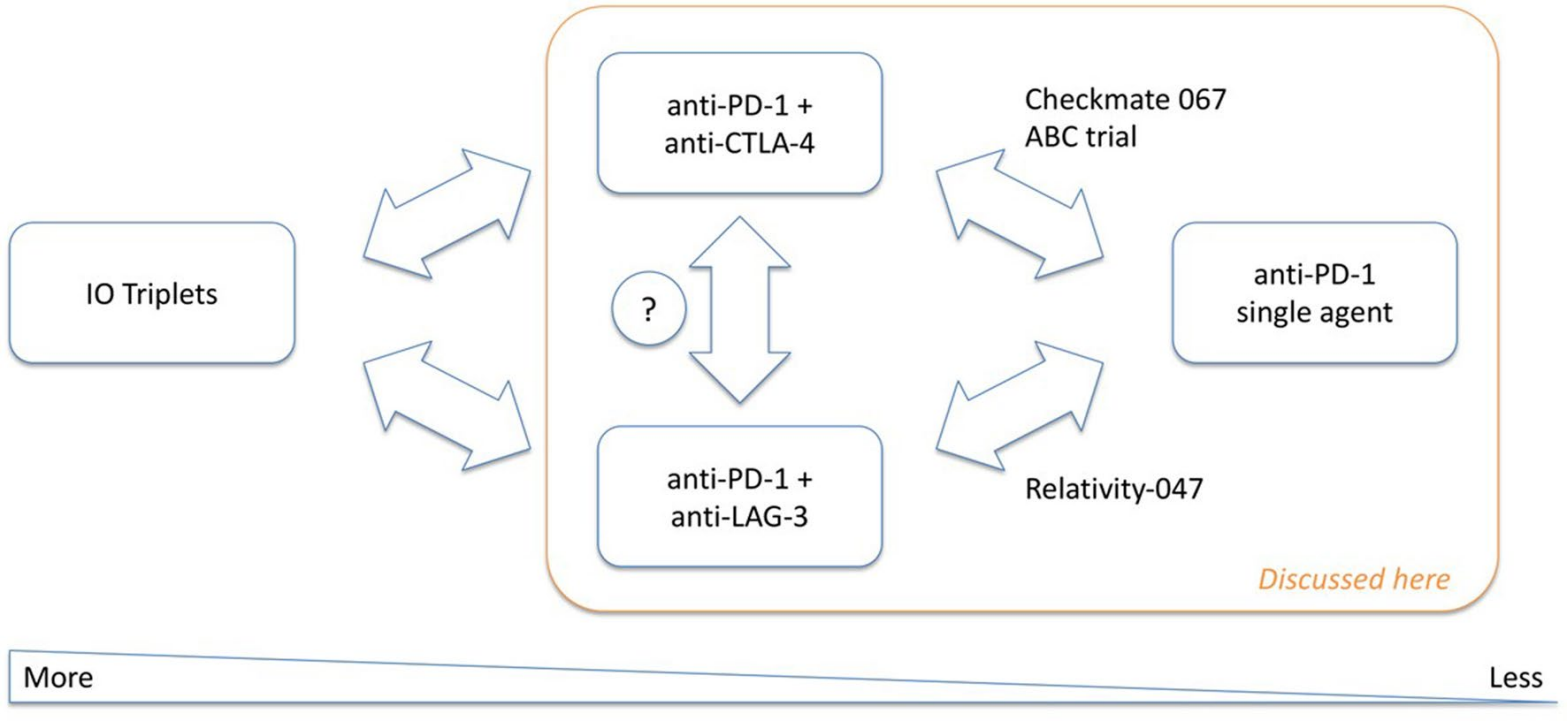
Overview and scope of the discussion “less is more”

An example of less is more was demonstrated by the combination of anti-PD-1 therapy with BEMPEG, a first-in-class CD122-preferential IL-2 pathway agonist. High-dose IL-2 has shown anti-tumor activity in patients with metastatic melanoma but with high rate of serious adverse events; BEMPEG was engineered to increase the half-life of IL-2 allowing for less frequent dosing and a more favorable safety profile. However, BEMPEG in combination with nivolumab showed no extra benefit regarding ORR, PFS, and OS versus nivolumab [44]. Both ORR and PFS were numerically lower in the arm including BEMPEG, although OS was similar, suggesting the addition BEMPEG might negatively affect outcomes. Significant limitations of the treatment include higher toxicity treatment cessation due to adverse events and use of systemic steroids that was higher in the BEMPEG arm. In addition, the effects of BEMPEG by creating conjugated version of IL-2 that shows preferential signalling to the dimeric form of the receptor and has limited interaction with the trimeric receptor on Teff may not be fully understood. Although BEMPEG design and validation was carefully carried out in vitro and in vivo, there is the possibility that the complex biology at the tumor site could trigger unexpected biological drug behaviour upon dissociation of the PEG moiety from the drug. The question is whether the IL-2 receptor is a valid target or whether the issues are related to BEMPEG. Other IL-2 derivatives are in clinical development and have favourable efficacy and toxicity profiles in the early clinical stages, although this was also true of BEMPEG. Early biomarkers of efficacy will be key to prioritize the transition from phase II into phase III clinical development in the future (Fig. 1).

### Role of adrenergic blockade in the immunotherapy efficacy

Both α- and β-adrenergic receptors are expressed by a variety of innate and adaptive immune cells. Although α-adrenergic receptors appear to provide stimuli for proinflammatory cytokine production during the classic acute stress response, β2-adrenergic receptors have been consistently observed to be the most highly expressed subtype on immune cells and are the exclusive adrenergic receptor expressed by T and B cells, suggesting they are a main mediator of catecholamine-induced immune regulation. In general, chronic stress via β-adrenergic signalling has been shown to promote an immunosuppressive immune profile.

Tumors can attract autonomic innervation which aids to tumorigenesis [45]. In melanoma, studies have shown that β2-adrenergic receptors are the major drivers in causing tumorigenic changes, and that the expression level of these receptors increase as disease progresses. Further, a variety of tumor types locally stimulate intratumoral norepinephrine production in states of stress leading to tumorigenic effects. In mouse models, enhanced tumorigenesis with β2-adrenergic receptor agonists (isoproterenol or terbutaline), reduced tumor growth when exposed to a non-selective β-adrenergic receptor antagonist (propranolol) and reversal of these pathways have been shown [46].

GLUT1 and glucose membrane transport proteins are critical for CD8+ T cell activation and effector function and these proteins are regulated by β2-adrenergic receptors. Expression of GLUT1 and the resultant glycolysis in CD8+ T cells undergoing activation is decreased by β2-adrenergic receptor blockade, showing a direct mechanism by which stressful stimuli can interfere with antitumor CD8+ T cell function. β2-adrenergic receptor blockade in mice exposed to chronic stress led to reductions in exhausted T cells and the expression of exhausted CD8+ T cell phenotypes (PD-1, LAG-3, TIM-3) on CD8+ TILs, enhanced metabolic activity and function of TILs, enhanced natural killer (NK) cells in the tumor microenvironment (TME), and suppressed tumor growth [47]. These findings are consistent with retrospective studies that have suggested that the incidental use of β-blockers is beneficial to patients with cancer receiving immunotherapy.

In a 3 + 3 dose escalation study for propranolol with pembrolizumab, nine patients with metastatic melanoma received increasing doses of propranolol in cohorts of 10, 20, and 30 mg twice daily [48]. No dose-limiting toxicities were observed, and ORR was 78%. The ratio of CD8 + T cell/monocytic myeloid-derived suppressor cells increased as compared to baseline in three patients on the highest propranolol dose, all of whom responded. Otherwise, no significant changes in treatment-associated biomarkers were detected, although there was an increase in IFN-α and a decrease in IL-6 in responders. This trial is continuing into phase II. The incorporation of pan-β-adrenergic blockade is a safe and highly cost-effective strategy that may enhance the efficacy of anti-PD-1 therapy with negligible additional toxicity.

### Genomics of therapy-resistant melanoma and translational opportunities

Genomic instability processes enable acquired resistance to targeted therapy. Understanding the structure and dynamics of these processes can help in the development of treatments that prevent rather than reverse resistance. One type of genomic instability is chromothripsis, which refers to a single cataclysmic event that generates extensive and complex genomic rearrangement of one or more chromosomes and has been found in many cancer types. Chromothripsis per se is not a mechanism of gene amplification, but during the process deletions can occur and pieces of DNA that drop out can circularize and form circular extrachromosomal DNAs (ecDNAs). As a result of non-Mendelian evolution, ecDNAs can exist in high copy numbers and lead to high gene amplification and extensive intratumoral heterogeneity. With prolonged selection, ecDNAs can re-integrate back into chromosomes and exist as intrachromosomal complex genomic rearrangement (CGRs) that amplify genes. Thus, ecDNAs and CGRs are well-suited to endow melanoma with variants that allow melanoma to escape from targeted therapy.

We have recently shown that ecDNA-and CGR-amplicons drive acquired mitogen-activated protein kinase (MAPK) inhibitor resistance in clinical *BRAF*^V600MUT^ and experimental (i.e., patient-derived xenografts) *NRAS*^MUT^ cutaneous melanoma. We showed further that ecDNAs/CGRs are likely derived from chromothripsis or shattering and restitching of chromosomal DNA. We found that the restitching of double-stranded DNA fragments into ecDNAs/CGRs is primarily driven by the double-stranded DNA break repair pathway, non-homologous end-joining. Importantly, targeting NHEJ by DNA-PK inhibitors in combination with MAPKi prevents acquired resistance by blocking the formation of ecDNAs/CGRs. This constitutes the scientific rationale for a phase 1b/2 trial design in *NRAS*^MUT^ melanoma.

### Trials to decrease toxicity and augment the benefit of combination checkpoint inhibition

IL-6 receptor blockade may decrease the incidence of irAEs. Expression of cytokines in colitis specimens from immune checkpoint inhibitor-treated patients with irAEs showed increased IL-6 expression in those colon tissues, and in tumors of non-responders versus responders [49]. Anti-CTLA-4 antibody added to IL-6 blockade augmented clinical benefit in murine solid tumor models, with increased CD8 + T cell tumor infiltration, T helper (Th) 1 cytokines, CXCL10, and CXCL11, and reduced infiltration of regulatory T cells (Tregs), Th17, macrophages, and myeloid-derived suppressor cells (MDSCs). Higher baseline serum IL-6 levels have also been associated with worse survival in patients receiving immune checkpoint blockade [50].

IL-6 blockade with tocilizumab has been shown to reverse steroid-refractory toxicity in checkpoint inhibitor-treated patients [51]. In order to assess if tocilizumab could reduce toxicity and/or augment efficacy of checkpoint inhibition, we designed a phase II trial of ipilimumab 1 mg/kg and nivolumab 3 mg/kg at ‘flipped doses’ with simultaneous IL-6 receptor blockade with tocilizumab 4 mg/kg every 6 weeks in a two-stage Simon design. In 28 patients who had started therapy, there were five irAEs [52]. Higher levels of baseline tumor necrosis factor (TNF)-α were associated with grade 3–4 toxicity. At median follow-up of 6 months, 14 of 20 patients had a response (70% ORR).

In another study, the safety and efficacy of tocilizumab 162 mg bimonthly plus ipilimumab 3 mg/kg and nivolumab 1 mg/kg was evaluated in 25 patients with melanoma. There was a trend to mitigate irAEs (11 patients with grade 3–4 irAEs), and ORR was 60% [53]. Biomarker analysis suggested that the dose of tocilizumab used may have been insufficient for full IL-6/Th17 pathway blockade.

In clinical trials of prophylactic TNF cytokine suppression with checkpoint inhibitor therapy, infliximab or certolizumab with standard dose ipilimumab 3 mg/kg plus nivolumab 1 mg/kg did not reduce toxicity [54]. There were 3/6 responders in the infliximab group, and 7/7 in the certolizumab group.

These data support the further investigation of IL-6 blockade and a phase II trial of ipilimumab plus nivolumab plus relatlimab with the IL-6 receptor blocking antibody sarilumab in patients with stage IV melanoma is planned.

### The role of neoantigen landscape in metastatic cancer patient response to pembrolizumab

Cytotoxic T cells are the major determinants of anti-cancer immunity. Anti-tumour T cell immunity is mediated by the physical interaction between T cell receptors and tumour antigens in complex with HLA. Tumor neoantigens resulting from the proteosomal degradation of variant proteins encoded by somatic non-synonymous mutations are promising targets for T cell therapy. Adoptive transfer of neoantigen-reactive T cells correlates with response to immunotherapy but the structural and cellular mechanisms of neoantigen recognition are not well understood.

**Fig. 2:**
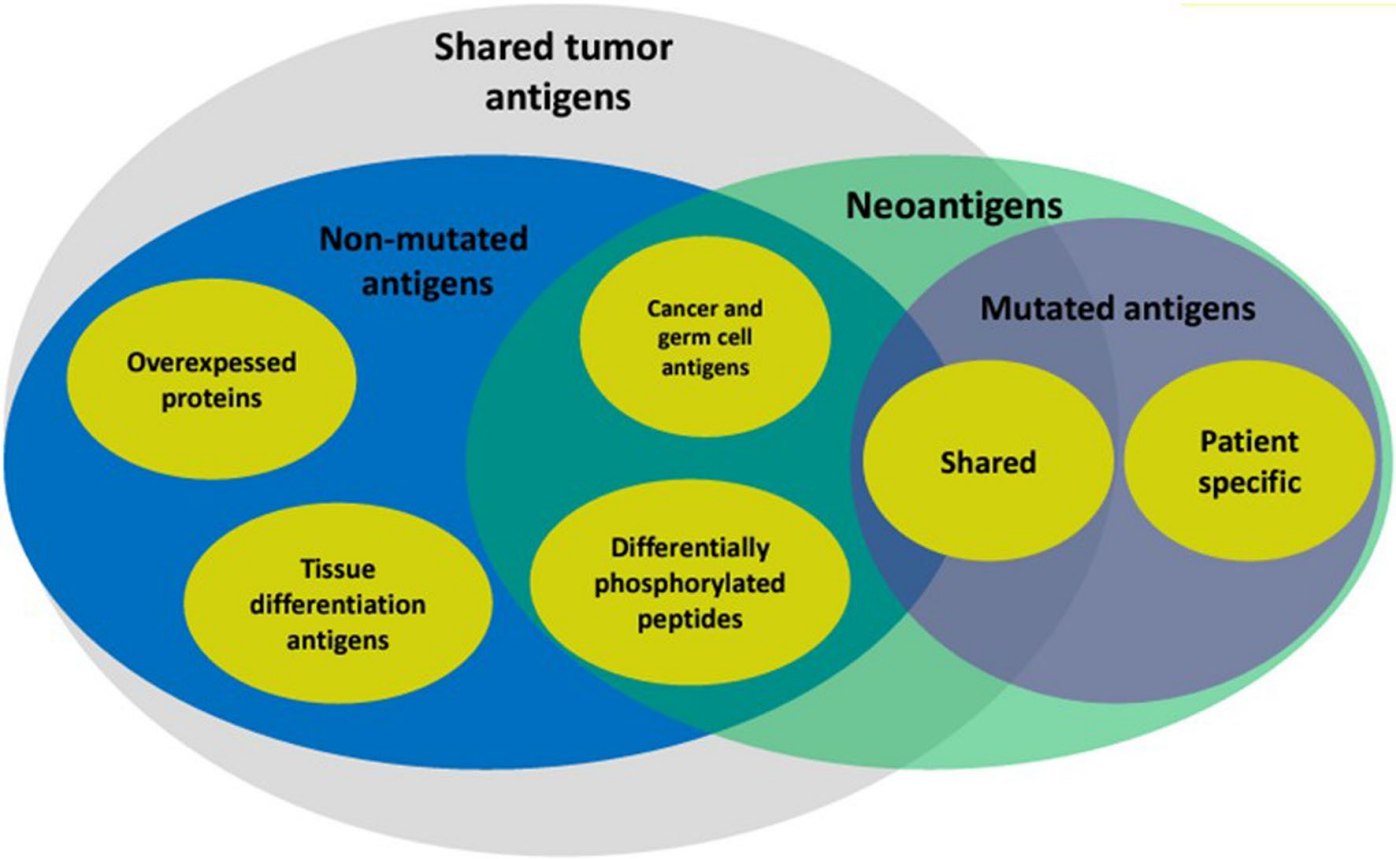
Type of tumor antigens

The B16F10 murine melanoma cell line is a common murine implantable tumour model of melanoma that is resistant to checkpoint blockade and has an elevated tumor mutational burden, including a number of driver genes. Vaccination with germline antigens (also known as cancer-testis) or neoantigens predicted and prioritized using genomic approaches elicit tumor specific delayed tumor growth. However, no melanoma B16 neoantigens have been thoroughly validated. We identified multiple cognate neoantigen:T cell receptor from B16F10, including a high affinity T cell receptor 47BE7, which targets the H2-Db-restricted neo-antigen Heat shock protein 2 (Hsf2 p.K72N68-76), that conferred specific recognition in B16F10 and exhibited effector function in vitro and in vivo [55]. Structural characterization revealed features of structural stability of the peptide-major histocompatibility class underlying enhanced immunogenicity of class II neoantigens. Hsf2 was the only melanoma B16 neoantigen that passed validation. Discovery of a fully validated neoantigen Hsf2 in melanoma B16 opens the opportunity to study the role of neoantigens in immunity of melanoma, including resistance to checkpoint blockade (Fig. 2).

## Emergent strategies

### Identification of high-risk melanoma patients with gene expression profiling assays

Gene expression profiling (GEP) is intended to predict recurrence or metastatic risk based on expression patterns of a selected panel of genes from the primary tumor and its use is becoming more prevalent. GEP development involves three stages, the first of which is discovery and involves gene identification, developing a training set, and lock-down of formula and cut-off point. The second stage is validation to evaluate GEP performance for its intended use and whether it can discriminate between high-risk and low-risk patients. Finally, clinical utility through impact and potential benefit on patient management, e. g. selecting high-risk patients for adjuvant treatment, is evaluated.

Three major GEP tests that have reported data in melanoma are MelaGenix, DecisionDx, and Merlin/SkylineDX. MelaGenix includes eight prognostic genes that correlate with survival. In a validation cohort of 245 patients with stage II cutaneous melanoma, high gene expression risk score was associated with decreased RFS, distant metastasis-free survival, and melanoma-specific survival [56]. In the NivoMela trial of stage II melanoma, MelaGenix is being used to select patients for randomization to adjuvant nivolumab treatment or observation. Another platform, DecisionDX, is a validated 31-GEP test to identify the risk of recurrence or metastasis including the likelihood of sentinel lymph node positivity for patients with stage I–III melanoma. The DecisionDx-GEP is now combined with patient clinicopathologic (CP) factors to further individualize prediction of risk of recurrence and sentinel lymph node biopsy positivity. A third platform, SkylineDX, based on identification of eight prognostic genes which correlate with SLNB metastasis also uses a CP-GEP model. In 210 patients with primary cutaneous melanoma who underwent sentinel lymph node biopsy, this CP-GEP model identified those at low risk for nodal metastasis [57].

The national Melanoma Prevention Working Group (MPWG) reviewed these three GEP tests in melanoma. Although each test has prognostic power, the MPWG did not support the routine use of GEP in the management of cutaneous melanoma patients at that point in time [58]. In the future, GEP might be able to help select appropriate patients for SLNB and adjuvant therapy.

### Melanoma induced metabolically deranged CD8+ T-cells are associated with immunotherapy resistance

Not all patients respond to immune checkpoint inhibitor therapy, highlighting a need for additional strategies including biomarkers to stratify patients for therapy. CD8 T cells have a critical function in response to immune checkpoint blockade but tumor-infiltrating lymphocytes (TILs), including CD8 T cells, express high levels of immune checkpoint receptors, e.g., PD-1, that can suppress cytotoxic T cell effector function. TILs can lose effector functions characterized by antigen unresponsiveness and reduced cytotoxic effector activity. Although checkpoint inhibitor blockade can revive effector T cell function, this may not be complete and may not explain lack of response in some patients.

Single-cell transcriptome comparisons between purified tumor-infiltrating CD8 T cells and peripheral blood lymphocytes (PBLs) from eight patients with melanoma identified several unique CD8 subpopulations [59]. Three overlapping clusters in CD8 TIL and PBL cells were identified, one of which had high levels of cytotoxic and exhausted markers, with high expression of multiple immune checkpoints, as well as increased levels of metabolic activities, specifically activated oxidative phosphorylation (OXPHOS). These high-OXPHOS CD8 T cells also had elevated levels of CD38/CD39 ectonucleotidases (CD8+ T_OXPHOS_ cells). Melanoma patients who did not respond to immune checkpoint inhibitor therapy had higher levels of these CD8+ T_OXPHOS_ PBLs and TILs which showed increased glucose metabolism, ATP production, and mitochondrial oxygen consumption. We developed a novel immunotherapy response predictive model focusing on CD8+ T_OXPHOS_ cells that can be assessed using either TILs or PBLs. In a training dataset, we predicted response to immune checkpoint inhibitor therapy in 11 of 12 patients. The model was then validated in four additional datasets, including one published dataset and three independent validation patient cohorts from our institution. In the validation cohorts, significantly higher non-response scores were seen in non-responders versus responders. These data demonstrated that the model has high predictive accuracy in TILs or PBLs. Given that non-invasive blood-based approaches have greater utility, the prediction platform was termed the non-invasive circulating T cells model (NiCir) [59].

### New therapeutic targets revealed through germline genetic polymorphisms

The T cell-inflamed and non-inflamed TME represent two categories of immune escape. Most responders to immunotherapy have T cell-inflamed tumors, characterized by high expression of chemokines, presence of CD8+ T cells, and a type I IFN signature, with immune escape primarily via inhibitory pathways. In comparison, non-T cell-inflamed tumors are characterized by a low inflammatory signature and the absence of intratumoral CD8+ T cells with immune escape via T cell exclusion. Anti-PD-1 efficacy is favoured by a T cell-inflamed TME and depends on interactions between Batf3+ DCs and CD8+ T cells within tumor sites. The T cell-inflamed TME is regulated by tumor cell-intrinsic oncogenic events, the composition of the commensal microbiota, and germline polymorphisms in immune regulatory genes. Interestingly, each of these dimensions can influence functionally important myeloid cells in the tumor microenvironment, e.g., β-catenin and decreased Batf3 DCs, gut microbiota and the shift from an M1 to M2 myeloid-derived suppressor cell phenotype, and germline PKCδ deficiency resulting in a shift to M1. Knowledge of these parameters is guiding opportunities for therapeutic intervention, including β-catenin inhibitors, microbiome manipulation through fecal microbial transplant or defined bacteria, and inhibitors targeting PKCδ.

### TIL Therapy in Melanoma: the future is now

Despite significant advances in the treatment of advanced melanoma through immunotherapy, most patients experience disease progression and there remains an unmet need to identify effective treatment for refractory disease. Adoptive cell therapy (ACT) with TILs is an immunotherapeutic strategy that harnesses the antitumor abilities of tumor-resident antigen-specific T cells. High response rates have been reported among patients with advanced melanoma after failure of an approved front-line therapy with TIL therapy. The expansion of cell therapy technologies has resulted in the establishment of off-site manufacturing facilities that have widened access to TIL therapy. Lifileucel, an autologous, centrally manufactured TIL product, has shown promise in patients with unresectable stage III-IV melanoma. In a phase II trial in 66 patients previously treated with multiple prior lines of therapy including immune checkpoint inhibitor therapy with or without BRAF ± MEK targeted agents, all of whom were refractory to anti-PD-1 therapy, ORR was 36% with two complete responses and 22 partial responses [60]. The disease control rate was 80%, and median duration of response was not reached after 18.7 months. The safety profile was consistent with known adverse events associated with nonmyeloablative lymphodepletion and IL-2; there was an expectedly high burden of adverse events related to IL-2 administration, followed by relatively few emergent adverse events after day 15 after infusion.

In the first randomized trial of TIL therapy, 168 patients with unresectable stage IIIC-IV melanoma, most of whom had progressed following anti-PD-1 treatment, were treated with ipilimumab or TIL therapy [61]. Patients treated with TIL therapy had significantly longer median PFS of 7.2 months compared to 3.1 months with ipilimumab. The ORR was 49% with TIL therapy versus 21% for ipilimumab, and median OS was 25.8 months and 18.9 months, respectively. Several other trials of TIL therapy are ongoing, including a pilot trial of lifileucel for melanoma brain metastases (NCT05640193).

### Preliminary immunological monitoring of first-in-human immunotherapy-trio of multivalent autophagosome vaccine, anti-GITR and anti-PD-1

Delayed PD-1 checkpoint inhibition following T-cell costimulatory agonist treatment (e.g., anti-OX-40) was found to be superior to the concurrent combination in cancer models, suggesting that premature PD-1 blockade can interfere with priming of new immune responses [62].

The combination of PD-1 inhibitor, multivalent DRibbles vaccine and GITR agonist has shown increased efficacy in preclinical models [63] and is being tested in a clinical trial of DRibbles vaccine ± GITR agonist, followed after two weeks by delayed anti-PD-1. DPV-001 (DRibbles vaccine) is an off-the-shelf multivalent autophagosome-enriched cancer vaccine that contains > 300 putative cancer antigens, including alternative cancer neoantigens—cancer’s dark matter, DAMPS, HSPs and agonists for TLR 2, 4, 7, 8, and NOD2 [64]. INCAGN01876 (GITR agonist) is a recombinant, humanized IgG1 κ monoclonal antibody that selectively binds to the extracellular domain of human GITR (CD357 or TNRSF18). Retifanlimab is a humanized, hinge-stabilized, anti-PD-1, IgG4 κ monoclonal antibody. In Arm 1 of the study, patients with recurrent or metastatic head and neck squamous cell carcinoma and good functional status, receive vaccine followed two weeks later by delayed anti-PD-1; while in Arm 2, patients receive combined vaccine/anti-GITR followed by delayed anti-PD-1. The primary endpoint of the study is safety, and secondary endpoints are objective response and survival. Biopsies are collected at baseline, weeks 12 and 24, with frequent blood sampling to allow exploratory correlative analyses.

### Dendritic cells and combinatorial adjuvants reprogram TME and lymphoid tissues for enhanced effectiveness of PD1 blockade

IL-12 production by DCs is needed for the induction of tumor-specific CTLs and Th1 cells, and the activation of NK cells. DC production of IL-12p70 predicts long-term outcomes in patients with different cancers who receive different DC vaccines [65–68]. High-IL-12 producing mature DCs can be induced by mediators of acute anti-viral immunity.

Type-1-polarized DCs induced by mediators of viral infections (αDC1s; included by the combination of type-1 and type-2 interferons with dsRNA, TNFα and IL-1β) preferentially interact with naïve, memory and effector T cells and induce CTL and Th1 cells against multiple cancer cells and tumor blood vessels antigens. In combination with dasatinib, an αDC1 vaccine targeting tumor blood vessel antigens induced durable clinical responses in checkpoint inhibitor-refractory melanoma [69]. The combination was well tolerated and resulted in immunologic and/or objective clinical responses in 6 of 13 (46%) evaluable patients. Clinical responses were associated with epitope spreading from vaccine antigens to lineage-specific antigens and the formation of tertiary lymphoid structures in the TME. Data from animal models and a pilot clinical trial indicate the ability of αDC1s to convert PD1-resistant cold tumors into PD-1-responsive ones.

Immune adjuvants induce intratumoral production of CTL attractants, but also Treg attractants and suppressive factors. However, combinatorial adjuvants can selectively induce CTL attractants and block Treg attractant CCL22 in myeloid cells. Rintatolimod (poly-I:C12U) can activate the TLR3 pathway and induce IFNα, ISG-60, and CXCL10 to promote CTL chemotaxis to ex vivo-treated tumors. without activating the MAVS/helicase pathway and avoiding NFκB- and TNFα-dependent induction of COX2, COX2/PGE2-dependent induction of IDO, IL10, CCL22, and CXCL12, and Treg attraction [70]. Induction of CTL attractants by rintatolimod was synergistically enhanced by exogenous IFN-α, which elevates TLR3 expression. The combination of αDC1 vaccine combined with a combinatorial chemokine-modulating regimen of rintatolimod, IFN-α2b and celecoxib, plus pembrolizumab is being assessed in a clinical trial in patients with PD-1-refractory HLA-A2 + melanoma.

### Next generation strategies to target MAPK signaling in cancer

RAS/MAPK signaling drives 30% of cancers and probably all melanomas. To date, MAPK-directed therapies have been successful in certain indications (i.e., BRAF V600E melanoma), encouraging in some (BRAF V600E colorectal cancer, KRAS G12C NSCLC), but have achieved only modest results in others. Clinical response is associated with MAPK inhibition in the tumor and acquired resistance is associated with lesions that reactivate MAPK more than other pathways. Adaptive mechanisms prevent potent and durable MAPK inhibition in the tumor and potential solutions, such as higher doses or additional drugs, are limited by toxicity.

Current RAF inhibitors (i.e., vemurafenib, dabrafenib, and encorafenib) preferentially bind to monomeric BRAF over dimeric BRAF. Consequently, RAF inhibitors are only effective in tumors in which monomeric BRAF contributes the totality of input to ERK. Development of adaptive drug resistance occurs when relief of negative feedback upon MAPK pathway inhibition results in the rapid formation of RAF dimers. RAF inhibitors equipotent for monomeric BRAF and dimeric BRAF have been developed but are predicted to have low therapeutic index, since inhibition of dimeric wild-type BRAF is likely to cause on-target toxicities in normal tissue.

A new class of RAF inhibitors are more selective for dimeric over monomeric RAF [71]. The difference in selectivity between equipotent and RAF dimer-selective inhibitors is through the restriction of the movement of the αC-helix of BRAF. Protein kinases exist in an equilibrium of active and inactive states, with the switch between them involving movements of two conserved structural motifs: the Asp-Phe-Gly (DFG)-motif and the αC-helix.

**Fig. 3:**
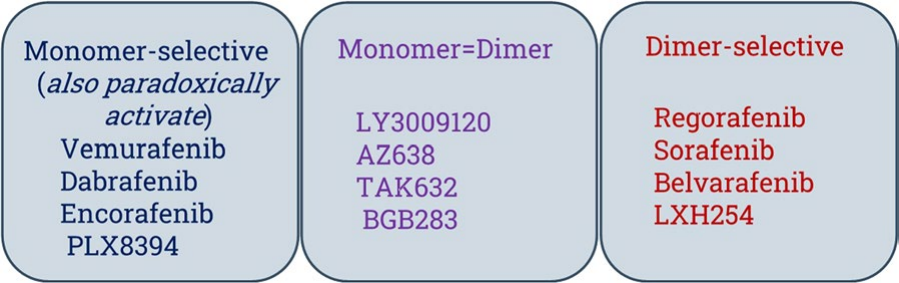
A taxonomy of RAF inhibitors

Dimeric RAF inhibitors in NRAS melanoma show some activity but mostly modest clinical efficacy. Transition of either wild-type BRAF or mutated BRAF (V600E) to the dimeric state increases its interaction with MEK. Current RAF inhibitor plus MEK inhibitor combinations may be limited by on-target toxicities caused by adverse synergy, in which certain MEK inhibitors enhance MAPK inhibition when in combination. A strategy of combining a RAF monomer-selective inhibitor with a RAF dimer-selective RAF inhibitor and a MEK inhibitor may help overcome adaptive resistance and retain a high therapeutic index when targeting BRAF (V600E) tumors. The triple combination of dabrafenib plus trametinib plus a RAF dimer-selective inhibitor resulted in more potent in vivo tumor suppression than dabrafenib plus trametinib in BRAF (V600E) therapy-resistant models [71]. Off-label use of the dual RAF inhibitor plus MEK inhibitor combination achieved durable responses in case reports of patients with colorectal cancer and multiple myeloma who had progressed on standard therapies [71, 72]. This MAPK inhibition triplet combination will be further assessed in patients with BRAF V600E mutated tumors (Fig. 3).

### Putting it all together—practical considerations for monday clinic

#### Does single agent immunotherapy have any role for the treatment of metastatic melanoma?

In the CheckMate 067 trial, PFS at 6.5 years was 34% with nivolumab plus ipilimumab versus 29% with nivolumab alone and 7% with ipilimumab alone [42]. Median OS in the combination group was 72.1 months, versus 36.9 months with nivolumab and 19.9 months with ipilimumab. In the RELATIVITY 047 trial, median PFS was significantly improved with nivolumab plus relatlimab compared to nivolumab monotherapy [43]. On the basis of these data, combination therapy seems preferable to monotherapy for all patients who are able to tolerate the additional toxicity and do not have a contraindication. First choice therapy should be anti-PD-1 combined with anti-CTLA-4, with nivolumab plus LAG-3 inhibitor relatlimab for patients who are not candidates for nivolumab plus ipilimumab.

#### Has the question of sequencing for patients with BRAF+ melanoma been put to rest?

The DREAMseq trial randomised 265 patients with BRAFV600-mutant metastatic melanoma to either the combination of nivolumab plus ipilimumab or dabrafenib plus trametinib, switching to the alternative combination at disease progression [73]. Patients who received nivolumab plus ipilimumab followed by dabrafenib plus trametinib was associated with significantly greater 2-year OS than the reverse sequence. Nivolumab plus ipilimumab was less effective as second-line therapy while second-line dabrafenib plus trametinib was a critical contributor to overall efficacy. Thus, combination immunotherapy followed by combination targeted therapy if necessary is the preferred option. A sandwich approach of targeted therapy for 8 weeks followed by immunotherapy until switching back to targeted therapy at disease progression, as investigated in the SECOMBIT trial, may also be of interest [74].

#### How should I approach adjuvant therapy for patients with stage II melanoma?

In the KEYNOTE 716 trial, adjuvant pembrolizumab significantly improved RFS and DMFS (both HR 0.64) versus placebo after 27 months of follow-up in patients with stage IIB or IIC melanoma [75]. The CheckMate 76K trial has also reported significantly improved RFS with nivolumab versus placebo (HR 0.42) in patients with completely resected stage IIB/C melanoma with standard wide local excision [76]. Grade 3–4 treatment-related toxicity occurred in 16% of pembrolizumab-treated patients and 10% of nivolumab-treated patients in these two studies. Based on these, adjuvant therapy may be offered to all patients with stage IIC disease and should be discussed as an option for patients with stage IIB disease. However, overtreatment with its associated toxicity and financial burden is still a concern.

#### Should I jump on the neoadjuvant bandwagon?

In the randomized phase II SWOG 1801 trial of patients with stage III–IV melanoma, neoadjuvant treatment with pembrolizumab resulted in a significant improvement in EFS compared with adjuvant pembrolizumab (HR 0.58) after a median follow-up of 14.7 months, with 2-year landmark EFS rates of 72% and 49%, respectively [19]. The neoadjuvant approach also improved OS (HR 0.63), although this was not statistically significant. These data are interesting and potentially practice changing; however, we need to wait for mature OS data. It may be that we are just treating patients earlier in their disease course and further clinical trial data are needed.

## Conclusions

The management of patients with advanced melanoma has advanced dramatically in the past decade, with the advent of immunotherapeutic and targeted approaches resulting in positive clinical outcomes for many patients beyond what was previously even considered possible. Treatments combining immunotherapy approaches with targeted therapies have further increased treatment efficacy and durability.

Despite these advances, many patients still experience primary and acquired resistance to treatment and toxicity that remain critical challenges. Thus, the focus in the field is on how best to address these challenges, through the development of novel treatments, new combination strategies, and new adjuvant or neoadjuvant approaches. The increasing complexity of the melanoma treatment landscape and the widening choice of treatment options requires new biomarkers and drug targets to improve accuracy in selecting appropriate therapy for individual patient.

Several predictive biomarkers of outcome, including tumor mutational burden, neoantigen load and pre-treatment CD8 lymphocyte count have been evaluated in clinical trials with different immunotherapies. Data also suggest that neoadjuvant therapy provides not only improved response as compared to adjuvant design, but it provides option to assess biomarkers for response. Pathological specimens provide a resource for correlative study to investigate tumor evolution, and the mechanism of resistance.

During the meeting, we summarized the current status of melanoma treatment landscape and outlined data from ongoing and new trials. Optimization of current therapies through rational combinations of existing and upcoming new drugs, drugs targets and biomarkers are the next stage in melanoma treatment with the ultimate goal to improve the efficacy and approaches for personalized therapy.

## Data Availability

Not applicable.

## Abbreviations

ACT: Adoptive cell therapy
BTLA: B- and T-lymphocyte-associated protein
CAR: Chimeric antigen receptor
CGR: Complex genomic rearrangement
CITE: Cellular indexing of transcriptomes and epitopes
CTL: Cytotoxic T lymphocyte
CTLA-4: Cytotoxic T-lymphocyte-associated antigen-4
DC: Dendritic cell
DLCBL: Diffuse large B cell lymphoma
ecDNA: Extrachromosomal DNA
ECT: Electrochemotherapy
EFS: Event-free survival
FDA: Food and drug administration
FFPE: Formalin-fixed paraffin-embedded
GEP: Gene expression profile
GITR: Glucocorticoid-induced tumour necrosis factor receptor-related protein
Hsf2: Heat shock protein 2
ICI: Immune checkpoint inhibitor
IFN: Interferon
IL: Interleukin
irAE: Immune-related adverse event
LAG-3: Lymphocyte-activation gene-3
MAPK: Mitogen-activated protein kinase
MDSC: Myeloid-derived suppressor cell
MPR: Major pathological response
MPWG: Melanoma Prevention Working Group
MSS: Microsatellite-stable
NK: Natural killer
NSCLC: Non-small-cell lung cancer
ORR: Overall response rate
OS: Overall survival
OXPHOS: Oxidative phosphorylation
PBL: Peripheral blood lymphocyte
pCR: Pathological complete response
PD-1: Programmed death-1
PD-L1: Programmed death ligand-1
PFS: Progression-free survival
pPR: Pathological partial response
RFS: Recurrence-free survival
SITC: Society of Immunotherapy for Cancer
SLNB: Sentinel lymph node biopsy
Th: T helper
TIL: Tumor-infiltrating lymphocyte
TIM-3: T cell immunoglobulin and mucin-domain containing-3
TKI: Tyrosine kinase inhibitor
TLND: Therapeutic lymph node dissection
TLR: Toll-like receptor
TMB: Tumor mutation burden
TME: Tumor microenvironment
TNF: Tumor necrosis factor
Tregs: Regulatory T cells
T-VEC: Talimogene laherparepvec
VISTA: V-domain immunoglobulin suppressor of T cell activation
